# The effects of using the PReDicT Test to guide the antidepressant treatment of depressed patients: study protocol for a randomised controlled trial

**DOI:** 10.1186/s13063-017-2247-2

**Published:** 2017-11-23

**Authors:** Jonathan Kingslake, Rebecca Dias, Gerard R. Dawson, Judit Simon, Guy M. Goodwin, Catherine J. Harmer, Richard Morriss, Susan Brown, Boliang Guo, Colin T. Dourish, Henricus G. Ruhé, Anne G. Lever, Dick J. Veltman, Anneke van Schaik, Jürgen Deckert, Andreas Reif, Michael Stäblein, Andreas Menke, Philip Gorwood, Géraldine Voegeli, Victor Pérez, Michael Browning

**Affiliations:** 1 P1vital Products Ltd, Howbery Park, Wallingford, Oxfordshire UK; 2 P1vital Ltd., Howbery Park, Wallingford, Oxfordshire UK; 3 Department of Health Economics, Center for Public Health, Medical University of Vienna, Vienna, Austria; 4 Department of Psychiatry, University of Oxford, Oxford, UK; 5 Oxford Health NHS Trust, Oxford, UK; 6 University of Nottingham, Nottingham, UK; 7 Department of Psychiatry, University of Groningen, University Medical Center Groningen, Groningen, The Netherlands; 8 Department of Psychiatry, VU University Medical Centre and Amsterdam Neuroscience, Amsterdam, The Netherlands; 9 Department of Psychiatry, Psychosomatics and Psychotherapy, University Hospital of Wuerzburg, Wuerzburg, Germany; 10 Department of Psychiatry, Psychosomatic Medicine and Psychotherapy, University Hospital Frankfurt, Frankfurt am Main, Germany; 11 Hôpital Sainte-Anne (CMME), CPN (UMR894), Université Paris-Descartes, Paris, France; 12 Universitat Autònoma de Barcelona, Barcelona, Spain

**Keywords:** Depression, Prediction, Treatment, Antidepressant, Primary care

## Abstract

**Background:**

Antidepressant medication is commonly used to treat depression. However, many patients do not respond to the first medication prescribed and improvements in symptoms are generally only detectable by clinicians 4–6 weeks after the medication has been initiated. As a result, there is often a long delay between the decision to initiate an antidepressant medication and the identification of an effective treatment regimen.

Previous work has demonstrated that antidepressant medications alter subtle measures of affective cognition in depressed patients, such as the appraisal of facial expression. Furthermore, these cognitive effects of antidepressants are apparent early in the course of treatment and can also predict later clinical response. This trial will assess whether an electronic test of affective cognition and symptoms (the Predicting Response to Depression Treatment Test; PReDicT Test) can be used to guide antidepressant treatment in depressed patients and, therefore, hasten treatment response compared to a control group of patients treated as usual.

**Methods/design:**

The study is a randomised, two-arm, multi-centre, open-label, clinical investigation of a medical device, the PReDicT Test. It will be conducted in five European countries (UK, France, Spain, Germany and the Netherlands) in depressed patients who are commencing antidepressant medication. Patients will be randomised to treatment guided by the PReDicT Test (PReDicT arm) or to Treatment as Usual (TaU arm). Patients in the TaU arm will be treated as per current standard guidelines in their particular country. Patients in the PReDicT arm will complete the PReDicT Test after 1 (and if necessary, 2) weeks of treatment. If the test indicates non-response to the treatment, physicians will be advised to immediately alter the patient’s antidepressant therapy by dose escalation or switching to another compound. The primary outcome of the study is the proportion of patients showing a clinical response (defined as 50% or greater decrease in baseline scores of depression measured using the Quick Inventory of Depressive Symptoms – Self-Rated questionnaire) at week 8. Health economic and acceptability data will also be collected and analysed.

**Discussion:**

This trial will test the clinical efficacy, cost-effectiveness and acceptability of using the novel PReDicT Test to guide antidepressant treatment selection in depressed patients.

**Trial registration:**

ClinicalTrials.gov, ID: NCT02790970. Registered on 30 March 2016.

**Electronic supplementary material:**

The online version of this article (doi:10.1186/s13063-017-2247-2) contains supplementary material, which is available to authorized users.

## Background

Depression is a common, serious and, in some cases, life‐threatening condition, affecting around 350 million people globally [1]. It is associated with significant socio-economic costs and has been predicted to become the greatest cause of disability worldwide by 2030 [2, 3]. In 2010, it was estimated that there were approximately 30 million patients with depression in Europe, with aggregated economic costs of approximately €92 billion [4]. Improvements in managing the treatment of depression are urgently needed to improve patient outcomes, contain rising healthcare costs, improve workplace productivity, and help to address global economic and societal challenges.

While a range of effective antidepressant medications are available, it takes 4–6 weeks after starting treatment before a physician can reliably detect whether the treatment is working [5]. Additionally, more than 50% of patients fail to respond to the first antidepressant treatment that they are prescribed [6, 7]. Therefore, it often takes several months to identify an effective antidepressant treatment for a majority of patients with depression. During this time a patient’s ability to work and function socially may be severely impaired.

Previous work has found that antidepressant medication alters the processing of emotional information [8]; for example, by causing depressed patients to label ambiguous facial expressions as more positive. Indeed, it has been argued that alteration of emotional processing bias represents one mechanism by which antidepressants cause improved mood in patients [8]. Consistent with this, the degree to which antidepressants induce these changes in emotional processing early in treatment is predictive of the later improvement in subjective symptoms as reported by depressed patients [9]. The PReDicT (Predicting Response to Depression Treatment) Test that will be used in this study has been developed from this work. The test combines a measure of emotional processing with the Quick Inventory of Depressive Symptoms – Self-Rated-16 (QIDS-SR-16) questionnaire [10]. The test has been designed to predict, after 1 week of antidepressant treatment, whether a patient will go on to respond to that treatment. A pilot study [11] of the measures used in the PReDicT Test found that it accurately predicted treatment response (defined as a 50% or greater reduction in baseline score on the QIDS-SR-16 by week 6 of treatment [6]) in 74% of depressed, primary-care patients.

The clinical opportunity provided by the early detection of antidepressant treatment response is that patients who are predicted to not be responding after 1 week can have their treatment altered at this time rather than waiting 4–6 weeks as is the case with present standards of care [5]. The current study will assess the effects of using the PReDicT Test to guide antidepressant treatment primarily in a primary-care setting. Depressed patients who are judged by their primary-care physician to require treatment with an antidepressant medication will be randomly assigned to have their treatment guided by the PReDicT Test (PReDicT arm) or to receive Treatment as Usual (TaU arm). Patients in the PReDicT arm will complete the PReDicT Test after one and, if necessary, 2 weeks of treatment. If the test indicates a likely non-response after either test, then patients will have their antidepressant treatment changed. Participants in the TaU arm will receive standard care, with subjective treatment response assessed after 4–6 weeks of treatment. The primary outcome of the study is the proportion of patients showing a response to treatment at week 8 of the study. Exploratory outcomes include assessment of treatment response at other time points and using alternative symptom measures.

If the PReDicT Test is successful in reducing the time needed to identify and initiate effective antidepressant therapy, it will be implemented routinely in primary care provided that it is cost-effective and the technology is acceptable for use by both patients and clinicians. Additional objectives of the study will, therefore, also include robust health economic analysis assessing cost-utility and cost-effectiveness as well as assessment of the barriers and facilitators to implementation of the PReDicT Test into primary-care services.

## Methods/design

The objectives of the study are listed in Table 1.

**Table 1:** Study objectives

Type of objective	Objective
Primary	To determine whether use of the PReDicT Test to direct antidepressant treatment results in an increased proportion of depressed patients showing a response (defined as 50% or greater reduction from baseline Quick Inventory of Depressive Symptoms – Self-Rated-16 (QIDS) score) to treatment at week 8 compared to TaU.
Secondary efficacy objective	To compare the change from baseline in QIDS scores (i.e. treated as a continuous variable) at week 8 between depressed patients receiving treatment directed by the PReDicT Test and those receiving TaU
	To determine whether use of the PReDicT Test to direct antidepressant treatment results in an increased proportion of depressed patients showing a response to treatment at week 8 compared to TaU, where response is defined as a decrease of 50% or more from baseline Montgomery-Åsberg Depression Rating Scale (MADRS) scores [10].
	To determine whether use of the PReDicT Test to direct antidepressant treatment results in an increased proportion of depressed patients achieving remission at week 8 compared to TaU where remission is defined as a QIDS score of 5 or less.
	To determine whether use of the PReDicT Test to direct antidepressant treatment results in an increased proportion of depressed patients achieving remission at week 8 compared to TaU where remission is defined as a MADRS score of 7 or less.
	To compare the change from baseline in QIDS score (i.e. treated as a continuous variable) at week 12 between depressed patients receiving treatment directed by the PReDicT Test and those receiving TaU.
	To compare the change from baseline in Quick Inventory of Depressive Symptoms – Self-Rated-16 (QIDS-SR-16) score (i.e. treated as a continuous variable) at 24 and 48 weeks between depressed patients receiving treatment directed by the PReDicT Test and those receiving TaU
Health economic objectives	To determine the impact on societal costs and cost-effectiveness/cost-utility of the PReDicT Test intervention in comparison to TaU over 24 weeks and over 48 weeks if feasible
Acceptability and implementation objectives	To explore how the PReDicT Tests are used by various stakeholders (patients, prescribing physicians and support staff), and the impact this has on care and care processes, in order to refine its future implementation across different countries.
Exploratory objectives	To compare the change from baseline in Generalised Anxiety Disorder Questionnaire, 7-item version (GAD-7) [11] score (i.e. treated as a continuous variable) at week 8 between depressed patients receiving treatment directed by the PReDicT Test and those receiving TaU.
	To compare the change from baseline on the depression and anxiety items (analysed separately) of the QIDS at week 8 between depressed patients receiving treatment directed by the PReDicT Test and those receiving TaU.
	To determine the change of cognitive function (assessed using the Digit Symbol Substitution Test (DSST) [12] ) from baseline to week 8 between depressed patients receiving treatment directed by the PReDicT Test and those receiving TaU.
	To compare the change from baseline in self-reported social and occupational functioning at weeks 8, 24 and 48 between depressed patients receiving treatment directed by the PReDicT Test and those receiving TaU.
Safety objective	To obtain evidence, as required by medical devices legislation, that the PReDicT Test is safe for use in primary care.

### Study overview

The study is a randomised, two-arm, multi-centre, open-label, clinical investigation of a medical device, the PReDicT Test. It will be conducted in depressed patients in five European countries (UK, France, Spain, Germany and the Netherlands). The Standard Protocol Items: Recommendations for Interventional Trials (SPIRIT) Checklist for the study is included as an additional file (Additional file 1) and the SPIRIT diagram for the study is included as Appendix 1: Table 2 and Appendix 2: Table 3.

### Study population

#### Inclusion criteria

Potential participants will be included if they meet the following criteria:

Male or female and aged between 18 and 70^1^ years inclusive.

Diagnosed with a depressive episode by a physician (either first episode or recurrent) and requiring treatment with a selective serotonin reuptake inhibitor (SSRI) medication (excluding fluoxetine due to its long half-life).

Prescribed an SSRI by a physician for the treatment of depression within the 7 days prior to visit 1, but has not yet started taking the medication.

Is intending to start SSRI treatment within 7 days of visit 1.

Willing and able to comply with study procedures.

Able to give written informed consent and has signed the Informed Consent Form prior to the first study-related procedure.

#### Exclusion criteria

Potential participants will be excluded if they meet the following criteria:

Previous history of mania.

Currently taking an antidepressant medication or has stopped antidepressant treatment within 2 weeks prior to visit 1.^2^

Requires immediate referral to alternative mental health services (e.g. where patient seen in primary care is referred to secondary-care services).

Presents to a physician with significant current suicidal intent requiring enhanced care.

Is employed directly by the investigator or is related to the investigator.

Currently taking part in another interventional clinical study which, in the opinion of the investigator, is likely to interfere with the objectives of the current study.

### Recruitment

Participants will be recruited from clinical services in the UK, France, Germany, Spain and the Netherlands. Potential participants with symptoms of depression will visit a physician. If a physician, using their standard practice, decides to prescribe an SSRI medication (excluding fluoxetine) to treat their patient’s depression, they can be considered for the study. Treatment with fluoxetine is excluded as this drug has a long half-life which limits the ability to rapidly switch to alternative medications as required in the current study if non-response is detected. The physician will provide a brief explanation of the study and will schedule a time for a screening and inclusion visit (see Appendix 1: Table 2) that can be cancelled if the participant decides not to take part in the study.

### Study centres

Details of study centres are provided at https://clinicaltrials.gov/ct2/show/NCT02790970. As the exact structure of the healthcare system, and the care pathway of depressed patients, varies by recruiting country, study centres were selected as the closest to primary-care settings in which antidepressant treatment is routinely managed. In the UK, Germany, Spain and the Netherlands this is in primary general-care settings; in France it is located in a secondary service to which primary-care physicians refer patients before initiating treatment.

### Study device

The PReDicT Test is a computer software application. It is CE-marked and is categorised as a Class I medical device. The PReDicT Test comprises the QIDS-SR-16 questionnaire, a facial recognition task [12] and a proprietary prediction algorithm. These have been developed and optimised to be sensitive to early changes in the negative emotional bias associated with depression.

The PReDicT Test will be accessed on a tablet, smartphone or personal computer connected to the Internet using a standard web browser. The participant will answer the questions and complete the tasks that appear on the screen. Analysis of the participant’s responses will indicate whether a treatment has successfully altered their emotional bias.

The PReDicT Test takes approximately 15 min to complete. Results, indicating response or non-response to treatment, are available immediately on completion of the test. Test results will only be provided to the study researcher/physician when appropriate and will not be provided to participants.

The PReDicT Test will be accessed through an electronic, patient-reported outcomes system (ePRO) which will also collect questionnaire data (e.g. QIDS-SR-16 score) and which will randomise participants and allow monitoring of participant compliance both with the PReDicT Test itself and with the trial procedures.

### Outcome measures

A summary of the outcome measures collected and their timings is provided in Appendix 1: Table 2.

*Primary* (*efficacy*): the primary outcome measure is the self-rated version of the Quick Inventory of Depressive Symptoms (QIDS-SR-16) [10]. This is collected at week 0 (baseline) and week 8 allowing calculation of the primary endpoint; whether the patient has shown a clinical response (defined as a greater than 50% reduction from baseline QIDS-SR-16 score by week 8) [6].

*Health economics*: a bespoke Health Economics Questionnaire (HEQ) will be used to measure direct and indirect costs throughout the study (NB a bespoke measure was developed as there is currently no validated measure which captures all relevant aspects of resource use and broader societal impacts). In addition, time directly related to the PReDicT Test (e.g. induction time, completing the PReDicT Test by participants, administering/reviewing the test by clinicians) will be recorded. The EQ-5D-5 L, a 5-dimensional, 5-level health-related quality of life questionnaire [13] will be collected as will the Oxford CAPabilities questionnaire-Mental Health (OxCAP-MH) to measure broader wellbeing [14].

*Acceptability and implementation*: bespoke Patients and healthcare providers Acceptability Questionnaires (PAQ) will be completed by all patients and healthcare providers in the study (NB these measures were developed during study development as no directly applicable measures were available). In addition, subsamples of patients and healthcare providers will complete bespoke semi-structured interviews relating to the acceptability and implementation of the PReDicT Test.

*Exploratory endpoints:* The Montgomery-Åsberg Depression Rating Scale (MADRS) [15] will be used to assess observer-rated symptoms of depression. The General Anxiety Disorder Questionnaire, 7-item version (GAD-7) [16] will be used to measure symptoms of anxiety in patients. The Digit Symbol Substitution Test (DSST) [17] will be used to measure cognitive functioning in patients and the ‘screener’ form for the Social Adjustment Scale, self-report version (SAS-SR) [18] will be used to assess quality of life.

*Safety endpoints:* all adverse events, adverse device events and device deficiencies will be collected up to the final study visit (week 8).

### Study design

The study is a randomised, two-arm, multi-centre, open-label, clinical investigation of a medical device, the PReDicT Test.

The study is divided into an 8- to 10-week clinical phase and a 40-week follow-up phase. Each participant will be in the study for a total of up to 48–50 weeks.

**Fig. 1:**
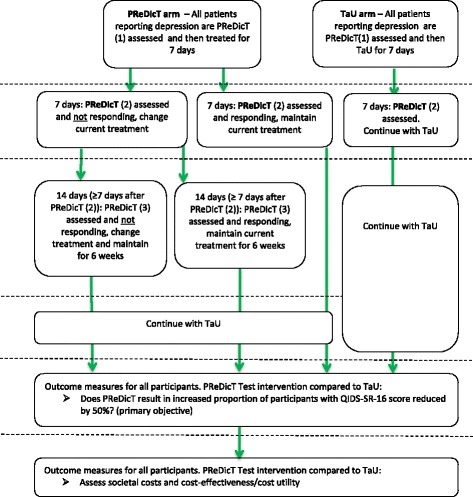
Outline of trial design

During the clinical phase, participants will attend between two and four study visits, depending on their study arm and their response to treatment. Some of these visits may be conducted by telephone. Participants will also complete weekly online questionnaires from home (see Fig. 1 and Appendix 1: Table 2 for time and events during this phase). During the follow-up phase participants will complete online questionnaires from home every 4 weeks over a 40-week period (see Appendix 2: Table 3 for time and events table).

*Clinical phase*: all participants will attend a screening visit. Those meeting the study entry criteria will complete the PReDicT Test during the visit and will be randomised to receive either treatment directed by the PReDicT Test (PReDicT arm) or TaU (TaU arm). Participants in the PReDicT arm will complete the PReDicT Test again at week 1. A change to participants’ antidepressant treatment will be suggested to the prescribing physician in accordance with locally appropriate guidelines and clinical judgement (either by dose escalation or switching to another drug) if the PReDicT Test indicates non-response to drug.^3^ In this case (i.e. the PReDicT Test at week 1 indicates a non-response), the participant will also complete the PReDicT Test at week 2. PReDicT Test results will be provided to the prescribing physician for participants in the PReDicT arm at weeks 1 and (if necessary) week 2.

Participants in the TaU arm will complete the PReDicT Test again at week 1.

PReDicT Test results will not be provided to the prescribing physician for participants in the TaU arm. Any change made to treatment by the prescribing physician will be based only on TaU (e.g. a change in drug treatment due to undesirable side effects or non-responsiveness based on clinical judgement).

At week 8, all participants will undergo clinical assessment of their response to treatment using questionnaires and assessments. They will also be asked to complete a Patient Acceptability Questionnaire (PAQ). A Health Economics Questionnaire (HEQ) will be also completed at weeks 0, 4 and 8.

*Follow-up phase:* starting at week 8, all participants will be asked to complete three online questionnaires every 4 weeks for 40 weeks. These questionnaires will capture depression status and health economic data. Two further questionnaires relating to social functioning and wellbeing will be completed at week 24 and again at week 48 of the study. Activities taking place during the online follow-up phase of the study are shown in the ‘time and events’ table in Appendix 2: Table 3.

Overall trial management and monitoring will be the responsibility of the study sponsor.

### Randomisation

Randomisation is carried out by the ePRO system using an independently validated programme.^4^ Study researchers will not be blind to participant group allocation. In order to reduce, as far as possible, the impact of expectation bias participants will not be explicitly told which study arm they are in, although they will be able to infer this if, for example, their treatment is changed after 1 week. The study cannot be blinded to a higher level as randomisation will determine the procedures that participants must complete.

Participants will be randomised into the PReDicT arm or the TaU arm using a 1:1 overall ratio. Randomisation will be stratified by study country and minimised by: (1) gender (male/female), (2) age (18–45 years and 45–70 years) and (3) baseline depression severity. Baseline depression severity will be calculated by the study software using QIDS-SR-16 scores. This will be categorised as mild/moderate (score 6–15) and severe/very severe (score of 16 or above).

The first 110 participants will be assigned to the PReDicT arm or the TaU arm in a 1:10 ratio. The rationale for predominantly recruiting into the TaU arm early in the study is that the first 100 participants will be used to further refine the algorithm used to guide treatment. During this phase minimisation will not be used. After this the minimisation procedure will be applied, with minimisation being followed in 66% of cases. This will result in a 1:1 overall ratio across the duration of the study.

### Statistical methods and data analysis

*Sample size*: sample size estimations suggest that 776 participants completed to week 8 are required.

The sample size was determined based on a minimum clinically relevant effect size (i.e. the difference in effect size between the TaU and PReDicT arms) which was set at 10% for the primary outcome (response at 8 weeks measured using the QIDS-SR-16). While there is no general agreement on minimum clinically relevant effect size, a review of the US Food and Drug Administration (FDA) data on antidepressant randomised controlled trials (RCTs) found that in RCTs of antidepressants versus placebo a minimum of 11% difference in response was significant and measurable [19]. In addition, during discussion with expert clinicians in the protocol development phase an effect size of less than 10% was considered to be clinically non-significant. The baseline response rate in the TaU group was estimated, based on data from a pilot study [11], at 40%. Adding the minimum clinically relevant effect of guided treatment of 10% to this value results in a response rate of 50% in the PReDicT-guided arm. Setting alpha at 0.05 and power at 80% indicates that a total sample size of 776 participants with primary outcome data (388 per group) would be required to detect the specified minimum clinically relevant difference of 10%. The expected attrition rate across the first 8 weeks of the trial, based on data from the pilot study, is 33%. Participants withdrawn from the study will not be replaced (that is an intention-to-treat analysis will be performed), thus 1158 participants will be enrolled to ensure that 776 participants complete the study.

*Analysis of primary objective*: logistic regression will be performed to explore treatment effects on the rates of response using an intention-to-treat approach.

*Analysis of exploratory objectives*: for the following outcomes which are analysed at week 8 of the study: MADRS, GAD-7, DSST scores, SAS-SR (screener version) and QIDS-SR-16 (including anxiety and depression items score), linear regression will be performed to quantify treatment effects with a baseline measure included as a covariate [20].

For other exploratory outcome measures, including SAS-SR (screener version) and QIDS-SR-16, which will be followed up monthly over the period of 1 year, multilevel modelling will be conducted to quantify treatment effects with ‘participant’ as a level-2 unit, treatment status and treatment × time interactions. Baseline measurement will be included in the multilevel models as a covariate [21].

For all regression modelling, age, gender, and baseline depression score will be included as covariates and country influence on treatment effect estimates will also be adjusted [22, 23]. Outcome missing value patterns will be examined and the influence of baseline measures and treatment allocation on missingness will investigated to inform multiple imputation procedure under the missing-at-random assumption.

An exploratory sensitivity analysis will test the effect of modifying the algorithm after 110 patients have completed the study.

*Analysis of health economic objectives*: the main health economic analysis will include: (1) a detailed patient-level cost analysis of health, social care and other broader societal costs for both the PReDicT and TaU arms of the study and (2) an incremental within-trial economic evaluation comparing the PReDicT arm and the TaU arm of the study in terms of their costs and outcomes over a 6-month follow-up period (week 0 to week 24). An optional health economic analysis may be also conducted over the full 12-month study follow-up period.

*Analysis of acceptability and implementation objectives*: acceptability questionnaires will be reported using descriptive statistics. Free text comments will be analysed thematically. Questionnaire and demographic data will be used to guide sampling for semi-structured interviews performed on a subsample of patients and clinicians.

Both the questionnaire and interview data will be used to develop an inductive analysis of the value and implementation of the PReDicT Test.

*Analysis of safety objectives*: safety data will be reported using descriptive statistics.

*Interim analysis*: an interim analysis will be performed when approximately 300 participants have been recruited. The interim analysis will assess recruitment rates within each country, adherence to the protocol and safety in terms of adverse events and device deficiencies in the two study arms. The interim analysis will also assess the effect size of the difference between the PReDicT arm and TaU arm on the primary outcome in order to assess the underlying assumptions of the power analysis and, if necessary, suggest an increased target recruitment figure. The interim analysis will be carried out and assessed by an independent Data Monitoring Committee (i.e. who are not authors of this paper or members of the study team) who will then, if necessary, make recommendations to the sponsor about whether alterations to the study protocol are required to meet the study objectives. The Data Monitoring Committee will consist of an experienced psychiatric trialist, an additional experienced trialist and a biostatistician.^5^ As all medications used in the study are licensed antidepressants, no formal stopping criteria will be used.

## Discussion

This trial provides a rigorous assessment of the clinical effectiveness as well as economic value and overall acceptability of deploying the PReDicT Test to guide antidepressant treatment of depressed patients. If successful, the trial will provide evidence to support a significant alteration in the standard treatment of depression. Specifically, it will suggest a method by which the process of identifying and initiating effective treatments can be substantially condensed relative to current standard practice [5]. By specifying non-responsive patients from a neuropsychological perspective, drawbacks of other studies of early switching of antidepressants might be overcome [24]. From the perspective of a depressed patient, a rational system which is able to more rapidly identify an effective treatment for their illness may well enhance confidence in, and improve adherence to, treatment plans. Conceivably, the ability to personalise treatment may also enhance the risk/benefit assessment of antidepressant use which has been questioned in a recent study which pooled data from clinical trials of the non-personalised use of antidepressants [25].

Clearly, the response of patients to antidepressant treatment is variable, with evidence from treatment trials indicating that separation from placebo can be detected at the group level after 1–2 weeks of treatment [26] and side effects commonly occurring early in the course of treatment. The PReDicT Test is, therefore, expected to be one of a number of potential sources of information that clinicians may draw on when making treatment decisions, rather than the sole arbiter of that decision. This is reflected in the design of the current trial in which clinicians are informed of the result of the PReDicT Test but are not required to use this information in their treatment decision if, for example, the patient has clearly responded or developed side effects.

The PReDicT Test used in this trial has been developed as part of a translational research programme which investigates treatment mechanism in depression, largely utilising non-clinical human models of cognition in controlled laboratory settings, [12]. This trial, therefore, also provides an important opportunity to test the potential benefits of using recent advances in cognitive neuroscience to improve patient care in mental health.

### Trial status

Recruitment started in the UK in July 2016. Recruitment in other countries is expected to start in late 2016/early 2017.

## Appendix 1

**Table 2:** Time and events table – clinical phase activities

	Visit 1: screening and PReDicT #1	Treatment start date	PReDicT #2	Visit 2 (if required)	Change of dose start date^a^ (if required)	PReDicT #3 (if required)	Visit 3 (if required)	Change of medication start date^b^ (if required)	Visit 4^c^
Requirements for timing of visit/event	Within 7 days of initial consultation	Day 0	≥7 days after day 0			≥ 7 days after changed dose			≥ 8–10 weeks after day 0
Preferred timing		Day 0	7–9 days after day 0	0–1 day after PReDicT #2	0–1 day after visit 2	7–9 days after changed dose	0–1 day after PReDicT #3	0–1 day after visit 3	8 weeks after day 0
Informed consent	X								
Entry criteria check	X								
Change in antidepressant (AD) medication agreed				X^a^			X^b^		
Review of concomitant medication									X
Review of AEs, ADEs and device deficiencies	X								X
Assessments									
PReDicT Test	X		X			X^a^			
QIDS-SR-16	X^d^	Completed weekly, starting 7 days after day 0							X^d^
Randomisation	X								
EQ-5D-5 L	X								X
HEQ	X								X
OxCAP-MH (UK and Germany only)	X								X
SAS-SR (screener version)	X								X
GAD-7	X								X
DSST	X								X
MADRS	X								X
Patient Acceptability Questionnaire									X

^a^If PReDicT #2 outcome = non-response, physician decides if antidepressant will be changed. PReDicT #3 will be completed

^b^If PReDicT #3 outcome = non-response physician decides if antidepressant will be changed

^c^Participants who withdraw will be invited to complete (in order of preference): visit 4 (at 8-week time point); visit 4 online questionnaires (from home at 8-week time point); Patient Acceptability Questionnaire (immediately); no additional data

^d^QIDS-SR-16 is included in the PReDicT Test

*AE* adverse event, *ADE* Adeverse Device Event, *EQ-5D-5 L* 5-dimensional, 5-level EuroQoL health-related quality of life questionnaire, *DSST* Digit Symbol Substitution Test*, GAD-7* Generalised Anxiety Disorder Questionnaire, 7-item version, *HEQ* Health Economics Questionnaire, *OxCAP-MH* Oxford CAPabilities questionnaire-Mental Health, *MADRS* Montgomery-Åsberg Depression Rating Scale, *QIDS* Quick Inventory of Depressive Symptomatology – Self Report

## Appendix 2

**Table 3:** Time and events table – follow-up phase activities

Online activity	Follow-up #1	Follow-up #2	Follow-up #3	Follow-up #4	Follow-up #5	Follow-up #6	Follow-up #7	Follow-up #8	Follow-up #9	Follow-up #10
Timing of activity^a^	Week 12 i.e. 4 weeks after visit 4	Week 16	Week 20	Week 24	Week 28	Week 32	Week 36	Week 40	Week 44	Week 48
QIDS-SR-16	X	X	X	X	X	X	X	X	X	X
EQ-5D-5 L	X	X	X	X	X	X	X	X	X	X
HEQ	X	X	X	X	X	X	X	X	X	X
OxCAP-MH (UK and Germany only)				X						X
SAS-SR (screener version)				X						X

^a^Data can be captured any time up to the due date of the next follow-up

*EQ-5D-5 L* 5-dimensional, 5-level EuroQoL health-related quality of life questionnaire, *HEQ* Health Economics Questionnaire, *OxCAP-MH* Oxford CAPabilities questionnaire-Mental Health, *MADRS* Montgomery-Åsberg Depression Rating Scale,*QIDS* Quick Inventory of Depressive Symptomatology – Self Report, *SAS-SR* Social Adjustment Scale, self-report version

## Additional file

Additional file 1: PReDiCT SPIRIT Checklist v0.2 6 Jan 2017. (DOCX 49 kb)

## Abbreviations

CE: Conformité Européene
DSST: Digit Symbol Substitution Test
ePRO: Electronic patient-reported outcomes
EQ-5D-5 L: 5-dimensional, 5-level EuroQoL health-related quality of life questionnaire
GAD-7: Generalised Anxiety Disorder Questionnaire, 7-item version
HEQ: Health Economics Questionnaire
MADRS: Montgomery-Åsberg Depression Rating Scale
OxCAP-MH: Oxford CAPabilities questionnaire-Mental Health
PReDicT Test: Predicting Response to Depression Treatment Test
QIDS-SR-16: Quick Inventory of Depressive Symptomatology – Self Report, 16 Item Version
SAS-SR (screener version): Social Adjustment Scale – Self-Report (screener version)
SSRI: Selective serotonin reuptake inhibitor
TaU: Treatment as Usual
UK: United Kingdom.

## Footnotes

1 Note an upper limit of 70 years was included as consultation with primary-care clinicians during study design suggested that older patients are often started on a reduced dose of antidepressants which is gradually titrated over the course of 1–2 weeks. This pattern of treatment would result in the PReDicT test being completed while a patient was on a potentially subtherapeutic dose.

2 A minimum 2 weeks absence from antidepressants was selected to ensure that previous treatment had a minimum effect on performance of the baseline PReDicT Test.

3 Note that a specific treatment algorithm was not imposed in this trial, rather the PReDicT Test was used to guide treatment decisions within the context of local practice.

4 Validation refers to the computerised systems validation process followed for the ePRO system which is designed to provide a framework for the design, development, acquisition, validation, implementation, release, support and maintenance of computerised systems, with specific emphasis on applications that are within scope of regulatory inspections, or are business critical. This process has been implemented in accordance with FDA Computerised Systems Used in Clinical Investigations May 2007 and FDA 21CFR Part 11 Electronic Records, Electronic Signatures 1997 and any subsequent amendments.

5 Members of the DMC are: DMC chair Prof. Hamish McAllister-Williams (Newcastle University), DMC member Dr. Peter Bower (University of Manchester) and DMC biostatistician Dr. Rhiannon Whitaker (Whitaker Research Ltd.).
